# Perceived Strengths and Gaps of Critical Care Fellows Across Emergency Medicine and Other Specialties

**DOI:** 10.5811/westjem.48854

**Published:** 2026-03-02

**Authors:** Lia Ilona Losonczy, Jordan Feltes, Jeremy B. Richards, Adam Odolil, Junfeng Sun, Aryana Kavuri, Mariam Hafez, Alisa Dewald, Nitin Seam

**Affiliations:** * George Washington University, Department of Emergency Medicine, Washinton, DC; † George Washington University, Department of Anesthesia and Critical Care, Washington, DC; ‡ George Washington University School of Medicine and Health Sciences, Washington, DC; § Harvard Medical School, Office of External Education, Boston, Massachusetts; || Western Atlantic University School of Medicine, Department of Medical Education, Freeport, The Bahamas; # National Institutes of Health Clinical Center, Critical Care Medicine Department, Bethesda, Maryland

## Abstract

**Introduction:**

Emergency physicians pursuing critical care training must enter fellowships designed for internal medicine, anesthesiology, or surgery trainees. In this study we aimed to assess how emergency medicine (EM)-trained fellows are perceived by critical care fellowship leadership compared to their peers and to identify specialty-specific strengths and gaps that may inform targeted educational approaches.

**Methods:**

We conducted a national, cross-sectional survey of program directors and associate/assistant directors of Accreditation Council of Graduate Medical Education-accredited critical care fellowships. Respondents rated the baseline competence of incoming fellows across 11 core critical care domains using a 5-point Likert scale. We compared competency ratings across residency training backgrounds using linear mixed models, accounting for clustering and adjusting for rater specialty where appropriate.

**Results:**

Of 429 distributed surveys, 118 (27.5%) were completed. Our respondents represented internal medicine-based fellowships (63, 53%), surgical fellowships (32, 27%), and anesthesia fellowships (23, 20%). On a 5-point Likert scale ranging from 1 = “Not competent” to 5 = “Very competent,” EM-trained fellows were rated significantly higher than their internal medicine-trained peers in intubation (3.93 vs 1.86, P < .01); vascular access (3.72 vs 2.52, P < .01); point-of-care ultrasound (3.80 vs 2.52, P < .01); surgical critical care (2.39 vs 1.99, P < .01); and neurologic emergencies (2.59 vs 2.10, P < .01). Fellows trained in internal medicine were rated higher in ventilator management (2.54 vs 2.06, P < .01); palliation (3.05 vs 2.08, P < .01); and renal physiology/acid-base disturbances (3.18 vs 2.40, P < .01). Slightly different patterns emerged when comparing EM to surgery and anesthesiology trainees, where EM-trained fellows were rated similarly or lower in procedural domains but demonstrated more robust competence in organ-specific physiology and ultrasonography. These patterns remained largely consistent in sensitivity analyses adjusting for rater specialty.

**Conclusion:**

Critical care fellows who trained in EM bring distinct strengths in diagnostics and resuscitation to critical care training, but their educational needs may differ from those of peers within specialty-specific fellowships. Tailoring curricula to address these differences can help ensure all trainees achieve proficiency across core domains.

## INTRODUCTION

Training in emergency medicine (EM) and critical care both require development of expertise in technical and cognitive skills in managing critically ill patients with diverse pathophysiology. Despite these commonalities, emergency physicians were ineligible to obtain board certification in critical care until 2011—decades after pathways for internal medicine, anesthesiology, and general surgery were established in the 1980s.^1^–^3^ As of 2024, 705 emergency physicians have completed fellowship programs and are board-certified in critical care, with many practicing as intensivists in academic institutions.^4^–^6^ However, there is no EM-specific pathway to critical care fellowship. Instead, EM residents seeking critical care training must choose from internal medicine, anesthesiology, or surgery fellowship programs. Because critical care training is designed for trainees from those backgrounds, these fellowships may emphasize skill development that does not fully align with the competencies of EM-trained fellows. Many EM-trained intensivists also split their time between the emergency department and the intensive care unit, which presents unique career challenges.^5^

While all critical care fellows must develop proficiency in core competencies to provide safe care,^7^–^9^ the emphasis on specific skills varies by specialty. Incoming fellows may have different strengths and weaknesses, necessitating individualized training approaches. Understanding how EM-trained intensivists compare with their peers in key domains upon entering fellowship is critical for refining training pathways and addressing potential gaps within current fellowship structures for these trainees.

### Importance

Identifying differences in competencies between EM-trained fellows and their colleagues trained in internal medicine, anesthesiology and surgery is essential for optimizing critical care curricula to train intensivists. Existing data do not suggest that EM-trained fellows in critical care medicine have lower graduation or board pass rates than their peers^10^; on the contrary, the data indicate comparable or even superior performance on standardized critical care exams. Thus, in this study our aim was not to remediate failure but to use these baseline differences in competency to advocate for fellowship curricula more thoughtfully tailored to the specific educational needs of EM trainees.

Prior literature has hypothesized what some of these differences in skillsets may be,^11^ but empirical evidence to date is lacking. Historically, EM representation in critical care was limited: only 12 emergency physicians completed critical care fellowships from 1974–1989; 15 from 1990–1999; and 43 from 2000–2007.^5^ But this number rose sharply to 190 between 2012–2016,^12^ reflecting substantial growth that began before, and accelerated after, the formal approval of EM eligibility for critical care medicine board certification in 2012. Given the increasing number of EM-trained intensivists and the ongoing intensivist shortage,^13^–^15^ understanding the educational needs of these trainees is essential. In this study we sought to understand program directors’ (PD) perceptions of baseline competence at fellowship entry rather than to determine whether current training structures fully meet those needs. Identifying these baseline differences can help fellowship programs tailor curricula more thoughtfully to the specific strengths and gaps of incoming EM fellows, ultimately supporting more effective training and patient care.

**Population Health Research Capsule**

What do we already know about this issue?

*Critical care fellows enter training from diverse residency backgrounds, but data comparing baseline competencies across specialties are limited*.

What was the research question?

*How do program leaders perceive baseline critical care competencies of emergency medicine (EM)-trained fellows compared with other specialties?*

What was the major finding of the study?

*The EM-trained fellows were rated more competent in point-of-care ultrasound and neurocritical care than all specialties, with other domains varying by specialty*.

How does this improve population health?

*Identifying baseline competency differences can inform targeted fellowship training, improving workforce readiness and quality of critical care delivery*.

### Goals of This Investigation

This is the first study to evaluate the baseline competence of incoming critical care fellows trained in EM compared to their counterparts in internal medicine, anesthesiology, and surgery. By surveying program leadership, we were better able to assess perceived strengths and weaknesses across key domains of critical care medicine in fellows from varied specialty backgrounds.

## METHODS

### Study Design and Setting

We distributed a cross-sectional survey to PDs and associate/assistant PDs of internal medicine-, anesthesiology-, and surgery-accredited critical care fellowships across the United States. Respondents assessed competency levels of their incoming fellows based on their primary residency training background (internal medicine, surgery, EM, and anesthesiology). We reiteratively reviewed the survey language until consensus was achieved on each specific question and how it was presented. Then, we pretested the survey with a cohort of representative respondents and subsequently modified it based on their feedback. It was then pilot-tested on the survey platform to review technical issues. The survey was then piloted with four academic intensivists from different clinical backgrounds, revised based on their feedback, and then re-piloted with an additional four academic intensivists who supervise trainees across multiple specialties. We obtained institutional review board approval from The George Washington University and informed consent from all respondents at the time of survey administration.

### Selection of Survey Participants

Eligible participants included PDs and associate/assistant PDs of critical care fellowships accredited by the Accreditation Council for Graduate Medical Education (ACGME). Inclusion criteria required respondents to have direct oversight of fellowship training. We obtained the list of programs that potentially accept EM applicants from the Emergency Medicine Residents Association Critical Care Fellowship database (https://www.emra.org/fellowships/critical-care-fellowships) and contacted PDs and associate PDs/program coordinators via an initial email followed by two reminder emails. Surveys were distributed electronically between Fall 2023–Spring 2024. Participation was voluntary, and data were collected anonymously to encourage candid responses.

### Measurements

Survey participants rated their incoming fellows’ competence in aggregate by specialty in 11 core areas of critical care based on ACGME-defined competencies that are standard across all specialty-specific intensive care fellowships. These domains include intubation, ventilator management, vascular access, sedation and analgesia, critical vasoactive medications, point-of-care ultrasound (POCUS), palliative care, perioperative critical illness, cardiovascular disease, renal failure/metabolic derangement, and neurologic emergencies. We instructed the respondents to select the level of competence they believed their incoming fellows to have in the 11 described domains. They were asked to rank each specialty in each domain on a 5-point Likert scale from “Not at all competent,” “Slightly competent,” and “Competent” to “Very competent and “Extremely competent.”

We calculated our response rate using the American Association of Public Opinion Research response rate 1 definition, which assumes all non-respondents are eligible and includes only fully completed surveys.^16^ Participants were not compensated for their time. See Appendix 1 for a copy of the survey.

### Outcomes

The primary outcome measure was PD-perceived competence of incoming fellows in the 11 predefined clinical domains. Secondary outcomes included comparisons between residency backgrounds and the influence of the program’s accrediting body (American Board of Anesthesiology [ABA], American Board of Internal Medicine [ABIM] American board of Surgery [ABS], or Standards Council of Canada) on competency ratings.

### Analysis

We analyzed data using linear mixed models to account for clustering of ratings from the same rater. Group means and differences of means were reported along with standard error. We adjusted multiple comparisons using the Dunnett method. Sensitivity analyses were conducted to assess the influence of respondent specialty on ratings. Reporting of this survey study was guided by the Consensus-Based Checklist for Reporting of Survey Studies.

## RESULTS

Of 429 surveys distributed, 118 (27.5%) were completed. Respondents included 93 (78%) PDs, 10 (8%) associate/assistant PDs, and 15 (13%) individuals in other program leadership roles. Programs were accredited through ABIM (63, 53%), ABS (32, 27%), and ABA (23, 20%). Sixty-three (53%) trained EM residents, 91 (77%) trained internal medicine residents, 65 (55%) trained surgery residents, and 49 (41%) trained anesthesiology residents (Table 1). The majority of the respondents supervised multiple different categories of incoming fellows; 50% supervised at least EM and internal medicine trainees, 32% at least EM and surgery, and 30% at least EM and anesthesia trainees, although there were a number who supervised trainees from three or even all four categories.

The PDs rated internal medicine-trained fellows lower than their EM-trained counterparts in intubation (1.86 vs 3.93, *P* < .01), vascular access (2.52 vs 3.72, *P* < .01), POCUS (2.52 vs 3.80, *P*< .01), surgical critical care (1.99 vs 2.39, *P* < .01), and neurologic emergencies (2.10 vs 2.59, *P* < .01). However, PDs rated IM-trained fellows higher in ventilator management (2.54 vs 2.06, *P* < .01), palliation (3.05 vs 2.08, *P* < .01), and renal physiology/acid-base disturbances (3.18 vs 2.40, *P* < .01). Slightly different patterns emerged when comparing EM trainees to surgery and anesthesiology trainees, where EM-trained physicians demonstrated less or similar competence in procedural skills but more robust competence in organ-specific physiology and in ultrasonography (Table 2).

As PDs may hold implicit biases that favor trainees from their own specialty—eg, anesthesiology PDs perceiving anesthesiology trainees as stronger in airway management than other PDs or internal medicine PDs viewing internal medicine trainees as more proficient in certain cognitive domains—we conducted a sensitivity analysis to examine whether such specialty-specific bias could meaningfully influence our findings. While the specialty of the evaluating PDs was found to be a statistically significant predictor (*P* < .05) in most domains, it did not qualitatively alter the competency comparisons between residency training backgrounds, other than to eliminate statistical significance of only a few of the differences where EM was noted to be marginally inferior. When accounting for the specialty of the respondent, there was no longer a statistically significant difference between internal medicine and EM trainees in ventilator management, or between EM trainees and either surgery or anesthesia trainees in vascular access. In all areas where EM trainees were considered more competent statistically, accounting for the respondent’s specialty did not modify that. Overall, this suggests that the observed differences in perceived competency are robust across different institutional perspectives.

## DISCUSSION

Our results indicate that EM-trained critical care fellows enter fellowship with a distinct skillset compared with their surgery, internal medicine, and anesthesiology-trained peers. Because this survey reflects perceived baseline competence at fellowship entry rather than the adequacy of existing training pathways, the findings should be interpreted as opportunities for early curricular adaptation.

Physicians trained in EM were consistently rated higher in POCUS and neurocritical care, compared to all specialties. They also demonstrated substantially greater perceived competence in airway management compared to internal medicine and surgical trainees. Differences of this magnitude on a 5-point Likert scale likely reflect meaningful variation in prior exposure and expected levels of supervision. For example, PDs rated incoming EM trainees as 3.93 in intubation—approaching “very competent”—compared with 1.86 for internal medicine trainees, falling between “not competent” and “slightly competent.” The insights of these PDs and supervisors suggest that EM and internal medicine fellows may require different levels of training in airway management when both are completing a critical care medicine fellowship, which is not currently the standard approach.

In contrast, EM trainees were rated lower in ventilator management, palliative care, and renal physiology—domains in which their peers from other specialties were perceived to have stronger baseline preparation. These findings complement the previously described predictions of EM-to-critical care medicine educational transitions, which also highlight differing baseline strengths and gaps across training backgrounds.^11^ Importantly, these patterns varied by comparison group, with EM trainees outperforming some specialties in selected domains while requiring more support in other domains when compared to others. The absence of a single consistent trend suggests that fellowship programs may benefit from recognizing residency-specific starting points rather than assuming uniform early proficiency across all incoming fellows.

Specialty-specific sensitivity analysis demonstrated that the PD’s own training background was a significant predictor of how they rated incoming fellows. Adjusting for the rater’s specialty eliminated statistical differences in a small subset of comparisons—such as ventilator management between internal medicine and EM trainees and vascular access between EM and surgery/anesthesiology trainees—while leaving most of the observed differences unchanged. Because the statistically significant differences favoring EM trainees persisted after adjustment, the pattern may suggest a modest bias in which EM trainees are viewed less favorably when rated by PDs trained in other disciplines. Nonetheless, most competency differences appear robust across institutional and specialty perspectives.

In addition to adjusting clinical rotations or didactic emphasis based on residency background, another potential strategy is the use of individualized, competency-focused assessments early in fellowship. Such assessments could help identify each fellow’s specific strengths and deficits at the beginning of training and enable fellowship programs to create truly individualized learning plans—an approach increasingly used in undergraduate and graduate medical education.^18^–^20^ Objective evaluations of procedural skills, diagnostic reasoning, ventilator management, and palliative care could also reduce the influence of specialty-related perceptual biases suggested by our sensitivity analysis.

Future research should evaluate whether individualized assessments improve educational efficiency, whether residency-specific curricular adjustments meaningfully close baseline gaps during fellowship, and how early competency differences correlate with performance outcomes, progression to independent practice, and standardized examination results. As the number of EM-trained intensivists continues to grow, especially within academic centers, this study raises an important question: Should an American Board of Emergency Medicine-specific critical care pathway be developed? Establishing a direct route for EM-trained physicians into critical care could ensure that training is tailored to their strengths while addressing gaps in their preparation. Further work would be needed to assess the feasibility, educational impact, and workforce implications of such a model.

## LIMITATIONS

This study has several limitations. First, we relied on subjective assessments from program leadership, which may have introduced bias based on preconceived notions of competency across training backgrounds. Respondents were not asked to directly compare trainees across residency backgrounds but instead rated the perceived baseline competence of the trainee groups they supervised. Comparisons across specialties were subsequently derived from these ratings. Program directors who did not train EM fellows were, therefore, unable to assess EM trainees directly; but they were able to provide assessments of the competence of their own incoming fellows. Second, the response rate was 27.5%, which, while inclusive of an appropriate distribution of critical care fellowships among the different specialties, may limit generalizability. Although 27.5% is below the median for single-institution trainee surveys, it falls within the published range (26–100%) for health professions-trainee surveys, of which multi-institution studies tended to have lower response rates. 17

Additionally, we used the minimum response rate definition, which is the most conservative estimate of the response rate.^16^ Because we specifically asked for a single respondent from an institution, we assumed that there would be few duplicates; however, since the data are de-identified, we cannot be 100% certain that there were not two respondents from the same institution in a small number of cases. Importantly, respondents were asked to report on their institution’s experience with trainees from different specialties rather than to evaluate individual trainees.

To address potential bias, we conducted a sensitivity analysis, which demonstrated that while the specialty of the program leadership influenced perceptions of fellow competencies, the overall trends in competency differences remained consistent, with the very specific exception that perhaps there was a bias toward EM residents appearing less competent when specialty bias was not adjusted for. Future studies incorporating objective competency assessments may help validate these results. Finally, because this study assessed perceived baseline competence rather than longitudinal learning outcomes, we could not determine whether existing critical care medicine fellowship curricula adequately meet EM trainees’ educational needs.

## CONCLUSION

Our findings underscore the unique skillset that EM-trained fellows bring to critical care training and highlight the areas where they may need additional support. While EM-trained physicians excel in some procedural skills such as intubation and point-of-care ultrasound, they also have the most competence in caring for neurological critical care patients; yet they require more training in areas such as ventilator management and palliative care. These differences do not easily fall into one category of needs, which suggests that critical care fellowship programs should consider implementing targeted, individualized learning strategies to optimize trainee development.

## Supplementary Information



## Figures and Tables

**Table 1:** Characteristics of 118 respondents by leadership role and specialty in a cross-sectional study of perceived competencies of incoming critical care trainees.

Category	Count (n)	Percentage of respondents
Leadership role		
Program director	93	78%
Assistant/associate program director	10	8%
Other leadership role	15	13%
Specialty of program		
ABIM-accredited	63	53%
ABS-accredited	32	27%
ABA-accredited	23	20%
Residency Specialties of Incoming fellows		
Emergency medicine	63	53%
Internal medicine	91	77%
Surgery	65	55%
Anesthesiology	49	41%

*ABIM*, American Board of Internal Medicine; *ABS*, American Board of Surgery; *ABA*, American Board of Anesthesiology.

**Table 2:** Perceived competence differences of incoming critical care fellows by residency background.

Trainee Group	Mean (SE)	Difference vs EM (SE)	Adjusted P-value
Intubation			
Emergency medicine	3.93 (0.12)	---------	---------
Anesthesiology	4.63 (0.13)	0.70 (0.17)	< .01
Internal medicine	1.86 (0.09)	−2.07 (0.14)	< .01
Surgery	2.02 (0.11)	−1.91 (0.15)	< .01

Comparison	Mean (SE)	Difference vs EM (SE)	Adjusted P-value

Ventilator Management			
Emergency medicine	2.06 (0.11)	---------	---------
Anesthesiology	3.16 (0.13)	1.10 (0.15)	< .01
Internal medicine	2.54 (0.10)	0.48 (0.12)	< .01
Surgery	2.29 (0.11)	0.23 (0.14)	.21
Vascular Access			
Emergency medicine	3.72 (0.12)	---------	---------
Anesthesiology	4.20 (0.14)	0.48 (0.17)	.02
Internal medicine	2.52 (0.10)	−1.20 (0.14)	< .01
Surgery	4.13 (0.12)	0.41 (0.15)	.02
Sedation/Analgesia			
Emergency medicine	2.79 (0.11)	---------	---------
Anesthesiology	3.90 (0.13)	1.11 (0.14)	< .011
Internal medicine	2.62 (0.10)	−0.18 (0.12)	.31
Surgery	2.57 (0.11)	−0.22 (0.13)	.23
Vasoactive Medications			
Emergency medicine	2.76 (0.10)	---------	---------
Anesthesiology	3.67 (0.11)	0.91 (0.13)	< .01
Internal medicine	2.95 (0.08)	0.19 (0.11)	.17
Surgery	2.81 (0.10)	0.05 (0.12)	.95
Point-of-care Ultrasound			
Emergency medicine	3.80 (0.11)	---------	---------
Anesthesiology	2.75 (0.13)	−1.05 (0.16)	< .01
Internal medicine	2.52 (0.09)	−1.28 (0.13)	< .01
Surgery	2.26 (0.11)	−1.54 (0.14)	< .01
Palliation			
Emergency medicine	2.08 (0.10)	---------	---------
Anesthesiology	1.98 (0.12)	−0.09 (0.14)	.85
Internal medicine	3.05 (0.09)	0.98 (0.11)	< .01
Surgery	2.60 (0.10)	0.53 (0.13)	< .01
Surgical Critical Care			
Emergency medicine	2.39 (0.11)	---------	---------
Anesthesiology	3.04 (0.12)	0.65 (0.15)	< .01
Internal medicine	1.99 (0.09)	−0.40 (0.12)	< .01
Surgery	3.59 (0.10)	1.20 (0.13)	< .01
Cardiac Critical Care			
Emergency medicine	2.73 (0.10)	---------	---------
Anesthesiology	2.88 (0.11)	0.15 (0.12)	.48
Internal Medicine	2.86 (0.08)	0.13 (0.10)	.47
Surgery	2.24 (0.10)	−0.49 (0.11)	< .01
Renal Physiology/Acid-Base Disturbances			
Emergency medicine	2.40 (0.10)	---------	---------
Anesthesiology	2.62 (0.11)	0.22 (0.12)	.20
Internal medicine	3.18 (0.08)	0.78 (0.10)	< .01
Surgery	2.60 (0.10)	0.20 (0.11)	.18
Neurocritical Care			
Emergency medicine	2.59 (0.10)	---------	---------
Anesthesiology	2.21 (0.12)	−0.38 (0.12)	< .01
Internal medicine	2.10 (0.09)	−0.49 (0.10)	< .01
Surgery	2.21 (0.10)	−0.38 (0.11)	< .01

Least square mean differences on 5-point Likert scale: 1 = “Not at all competent”, 5 = “Extremely competent.”

*EM*, emergency medicine; *SE*, standard error.
