# Supplementary material for: Perceived Strengths and Gaps of Critical Care Fellows Across Emergency Medicine and Other Specialties
Source: West J Emerg Med. 2026 Mar 2;27(2):483–9. doi: 10.5811/westjem.48854 (PMC13016041; doi:10.5811/westjem.48854)
Supplement: Supplementary file 1 [file wjem-27-483-s001.pdf]

Critical Care Fellowship Incoming Competence Survey

Please complete the survey below. Thank you!

Please characterize your fellowship program (select all that apply):	☐ Surgical Critical Care (ABS)<br>☐ Critical Care Medicine (ABIM)<br>☐ Anesthesia Critical Care Medicine (ABA)
Please indicate your role within the fellowship program (choose one):	○ Program Director<br>○ Assistant or Associate Program Director<br>○ Other
Your role in the fellowship program:	__________________________________
Please indicate from which residency training programs your fellowship program accepts applicants (select all that apply):	☐ Internal Medicine (IM)<br>☐ Emergency Medicine (EM)<br>☐ Anesthesia<br>☐ General Surgery<br>☐ Internal Medicine subspecialty fellowships (for example, fellows who already completed cardiology, nephrology, infectious diseases)<br>☐ Other
Please indicate from which OTHER residency training programs your critical care fellowship accepts applicants:	__________________________________
Please select all of the primary training pathways (i.e. prior residencies) for the critical care fellows you supervise clinically, even if they are not directly in your program.	☐ Internal Medicine (IM)<br>☐ Emergency Medicine (EM)<br>☐ Anesthesia<br>☐ General Surgery<br>☐ Other
Please indicate which OTHER residency training programs the critical care fellows you supervise come have come from:	__________________________________

For the following 11 questions, please select the level of competence you believe your INCOMING FELLOWS have based on their pre-fellowship residency training program in the following domains. These domains are considered fundamental to critical care training, and they were selected as they represent areas in the ACGME program requirements across CCM, SCC, and ACCM fellowships.

1. Procedural skills of intubating critically ill patients:

	Not At All Competent	Slightly Competent	Competent	Very Competent	Extremely Competent	N/A
EM Trained Fellows	○	○	○	○	○	○
IM Trained Fellows	○	○	○	○	○	○
Surgery Trained Fellows	○	○	○	○	○	○
Anesthesia Trained Fellows	○	○	○	○	○	○

Please select the level of competence you believe your INCOMING FELLOWS have regarding:

2. Ventilator management for critically ill patients:

	Not At All Competent	Slightly Competent	Competent	Very Competent	Extremely Competent	N/A
EM Trained Fellows	○	○	○	○	○	○
IM Trained Fellows	○	○	○	○	○	○
Surgery Trained Fellows	○	○	○	○	○	○
Anesthesia Trained Fellows	○	○	○	○	○	○

Please select the level of competence you believe your INCOMING FELLOWS have regarding:

3. Advanced vascular access ("vascular access" refers specifically to central venous catheters and arterial lines, and does not include peripheral IV placement, pulmonary artery catheter placement, IO placement, or PICC line placement)

	Not At All Competent	Slightly Competent	Competent	Very Competent	Extremely Competent	N/A
EM Trained Fellows	◯	◯	◯	◯	◯	◯
IM Trained Fellows	◯	◯	◯	◯	◯	◯
Surgery Trained Fellows	◯	◯	◯	◯	◯	◯
Anesthesia Trained Fellows	◯	◯	◯	◯	◯	◯

Please select the level of competence you believe your INCOMING FELLOWS have regarding:

4. Sedation/analgesia in critical care setting:

	Not At All Competent	Slightly Competent	Competent	Very Competent	Extremely Competent	N/A
EM Trained Fellows	○	○	○	○	○	○
IM Trained Fellows	○	○	○	○	○	○
Surgery Trained Fellows	○	○	○	○	○	○
Anesthesia Trained Fellows	○	○	○	○	○	○

Please select the level of competence you believe your INCOMING FELLOWS have regarding:

5. Critical vasoactive medications (vasopressors/inotropes/antiarrhythmics) in the critical care setting:

	Not At All Competent	Slightly Competent	Competent	Very Competent	Extremely Competent	N/A
EM Trained Fellows	○	○	○	○	○	○
IM Trained Fellows	○	○	○	○	○	○
Surgery Trained Fellows	○	○	○	○	○	○
Anesthesia Trained Fellows	○	○	○	○	○	○

Please select the level of competence you believe your INCOMING FELLOWS have regarding:

6. Point of care ultrasound in critically ill patients:

	Not At All Competent	Slightly Competent	Competent	Very Competent	Extremely Competent	N/A
EM Trained Fellows	○	○	○	○	○	○
IM Trained Fellows	○	○	○	○	○	○
Surgery Trained Fellows	○	○	○	○	○	○
Anesthesia Trained Fellows	○	○	○	○	○	○

Please select the level of competence you believe your INCOMING FELLOWS have regarding:

7. Palliative care and management of end of life transitions including disease-appropriate and patient-oriented pain, anxiety, and distress management techniques, as well as goals of care discussions with patients and family.

	Not At All Competent	Slightly Competent	Competent	Very Competent	Extremely Competent	N/A
EM Trained Fellows	○	○	○	○	○	○
IM Trained Fellows	○	○	○	○	○	○
Surgery Trained Fellows	○	○	○	○	○	○
Anesthesia Trained Fellows	○	○	○	○	○	○

Please select the level of competence you believe your INCOMING FELLOWS have regarding:

8. Clinical management of perioperative/surgical critical illness:

	Not At All Competent	Slightly Competent	Competent	Very Competent	Extremely Competent	N/A
EM Trained Fellows	○	○	○	○	○	○
IM Trained Fellows	○	○	○	○	○	○
Surgery Trained Fellows	○	○	○	○	○	○
Anesthesia Trained Fellows	○	○	○	○	○	○

Please select the level of competence you believe your INCOMING FELLOWS have regarding:

9. Management of cardiovascular disease in critical care (ie, arrhythmias, heart failure, valvular dysfunction, hypertensive emergencies)

	Not At All Competent	Slightly Competent	Competent	Very Competent	Extremely Competent	N/A
EM Trained Fellows	○	○	○	○	○	○
IM Trained Fellows	○	○	○	○	○	○
Surgery Trained Fellows	○	○	○	○	○	○
Anesthesia Trained Fellows	○	○	○	○	○	○

Please select the level of competence you believe your INCOMING FELLOWS have regarding:

10. Management of renal failure, acid/base disturbances, and critical electrolyte abnormalities.

	Not At All Competent	Slightly Competent	Competent	Very Competent	Extremely Competent	N/A
EM Trained Fellows	○	○	○	○	○	○
IM Trained Fellows	○	○	○	○	○	○
Surgery Trained Fellows	○	○	○	○	○	○
Anesthesia Trained Fellows	○	○	○	○	○	○

Please select the level of competence you believe your INCOMING FELLOWS have regarding:

11. Management of neurologic emergencies and/or the neuro critical care patient (ie, status epilepticus, acute CVA and ICH, cerebral edema, brain death. Does not include procedural skills)

	Not At All Competent	Slightly Competent	Competent	Very Competent	Extremely Competent	N/A
EM Trained Fellows	○	○	○	○	○	○
IM Trained Fellows	○	○	○	○	○	○
Surgery Trained Fellows	○	○	○	○	○	○
Anesthesia Trained Fellows	○	○	○	○	○	○
